# Risk Factors for Acute Hemorrhagic Rectal Ulcer Syndrome and Its Prognosis: A Density Case-Control Study

**DOI:** 10.1155/2018/8179890

**Published:** 2018-08-08

**Authors:** Toshihiko Komai, Fumio Omata, Yasutoshi Shiratori, Daiki Kobayashi, Hiroko Arioka

**Affiliations:** ^1^Department of Internal Medicine, St. Luke's International University, 9-1 Akashi-cho, Chuo-ku, Tokyo 104-8560, Japan; ^2^Department of Allergy and Rheumatology, Graduate School of Medicine, The University of Tokyo, 7-3-1 Hongo, Bunkyo-ku, Tokyo 113-8655, Japan

## Abstract

Acute hemorrhagic rectal ulcer syndrome (AHRUS) can cause fatal gastrointestinal bleeding. However, there have been few epidemiological studies investigating risk factors of AHRUS. To determine the risk factors and predict one-year survival after onset of AHRUS, we conducted a retrospective density case-control study in a tertiary referral hospital. Patients with hematochezia, bloody stool, and rectal ulcer confirmed by colonoscopy between 2003 and 2011 were diagnosed as AHRUS (*n* = 38). Patients with malignancies, infectious colitis, ulcerative colitis, or solitary rectal ulcer syndrome were excluded. Control subjects (*n* = 123) without rectal ulcer were selected by risk set sampling for each AHRUS. Multivariate logistic regression analyses revealed that the significant adjusted odds ratio (95% confidence interval) of hospitalization, antithrombotic drug use, and one gram increase of serum albumin was 15.7 (2.25–108.9), 12.1 (1.53–94.4), and 0.11 (0.02–0.52), respectively. Endoscopic hemostasis for rectal bleeding was performed in 8 cases (21%). Seventeen percent of patients died within one year after the episode of AHRUS from non-AHRUS causes. This study revealed that hospitalization, antithrombotic drug use, and lower serum albumin value were significant risk factors for AHRUS, and that AHRUS was an unfavorable prognostic condition. This information could be helpful in recognizing high-risk patients of rectal bleeding and applying preventive measures.

## 1. Introduction

Rectal ulcer, unrelated to malignancy, inflammatory bowel diseases (IBD), or infectious colitis includes two distinct disease entities: solitary rectal ulcer syndrome (SRUS) [1, 2] and acute hemorrhagic rectal ulcer syndrome (AHRUS) [3]. SRUS is a chronic benign disorder, most common in young adults, often associated with bowel disturbances, abnormal defecation, and mucosal prolapse [4, 5]. AHRUS is characterized by sudden massive rectal bleeding, most often in elderly patients with underlying comorbidities [6, 7]. AHRUS has been reported to be the most common cause of acute lower gastrointestinal bleeding in hospitalized patients with comorbidities [3, 6, 7].

Fecal evacuation disorder was reported to be a potential risk factor for SRUS [8–10], and gut-directed biofeedback therapy is an effective behavioral intervention [8, 9]. However, there have been few studies investigating risk factors and prophylactic interventions of life-threatening AHRUS. This lack of information may contribute to delay in making the diagnosis and in instituting preventive measures. In addition, there has been no report of survival analysis of the patients suffering from AHRUS. The aims of our study were to determine risk factors of AHRUS and to predict its one-year survival.

## 2. Materials and Methods

### 2.1. Study Population

A total of 23,988 colonoscopies were performed in a tertiary referral hospital in Tokyo, Japan, from 2003 to 2011. Thirty-eight cases with the diagnosis of AHRUS [3, 6] were identified after excluding associated ulcerative colitis (*n* = 3), infectious colitis (*n* = 1), rectal ulcer with no gastrointestinal bleeding (*n* = 4), SRUS (*n* = 1), and lack of laboratory data (*n* = 2). Subjects who had not given informed consent for use of their electronic records and subjects under 20 years of age were also excluded. We waived informed consent from patients who were included in our study.

**Figure 1:**
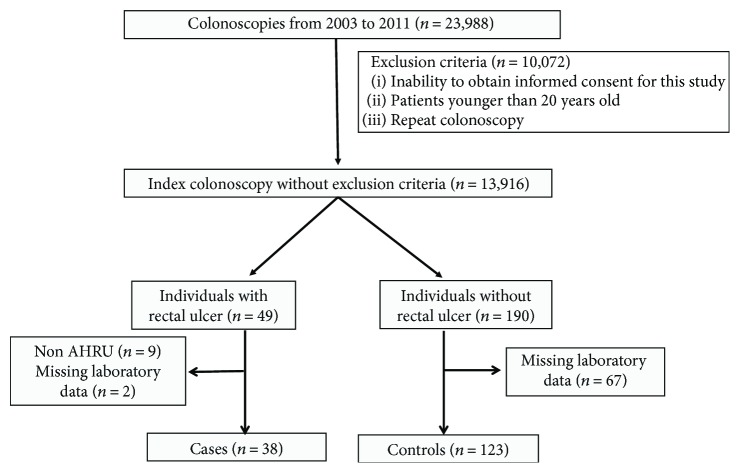
Study flow diagram depicting selection of cases and controls. 13,916 index colonoscopies out of 23,988 were performed in the patients older than 20 years of age in a tertiary medical center in Tokyo, Japan. Thirty-eight acute hemorrhagic rectal ulcer syndrome patients were diagnosed. From the same database of index colonoscopies, 123 patients without rectal ulcer were selected as a control group by risk set sampling.

One hundred twenty-three subjects without rectal ulcer and with adequate laboratory values were selected as controls by risk set sampling [11, 12] (Figure 1). When the laboratory values of controls were missing on the same day of colonoscopy, their laboratory values within 6 months after colonoscopy were imputed. This study was approved by St. Luke's International University Research Ethics Committee (authorization number: 11-R162).

### 2.2. Data Availability

The data of this study were handled with all of the authors under strict control, and availability was restricted for ethical reasons. However, anonymized data could be available for other researchers upon request with the permission of our ethical committee.

### 2.3. Diagnosis of Rectal Ulcer

The diagnosis of rectal ulcer in cases and confirmation of no rectal ulcer in controls were established with colonoscopy. AHRUS was defined as ulcer associated with hematochezia or bloody stool, whereas rectal ulcer was present at colonoscopy after excluding other diseases which might cause rectal ulcer [3, 6]. The location of ulcers in the rectum was classified like that used for colorectal cancer: rectosigmoid (Rs), rectum above the peritoneal reflection (Ra), or rectum below the peritoneal reflection (Rb).

### 2.4. Candidate Risk Factors for AHRUS

We investigated whether age, gender, comorbidities, laboratory values, hospitalization, and antithrombotic use were associated with AHRUS.

### 2.5. Statistical Analyses

Fisher's exact test was applied for proportion. Student's *t*-test or Wilcoxon rank sum test was used for continuous variables. Bivariate and multivariate logistic regressions were used to calculate odds ratio (OR). Variables with *P* value less than 0.2 in bivariate logistic regression were used in multivariate analyses. *P* value less than 0.05 was considered statistically significant. Kaplan-Meier estimates were used for survival analysis. All 95% confidence intervals were two-sided. All analyses were conducted with JMP® version 13 statistical software (SAS Institute, Cary, NC). In conducting this study, we followed the checklist of items for case-control study described in strengthening the reporting of observational studies in epidemiology (STROBE) statement [13].

## 3. Results

### 3.1. Characteristics of Patients with AHRUS

Table 1 presents patients' characteristics, candidate risk factors for AHRUS, laboratory data, and the results of bivariate analyses. Age, hospitalization, antithrombotic drug use, comorbidities (hypertension, ischemic heart disease, and cerebrovascular disease), and laboratory findings (serum albumin, serum creatinine, white blood cell count, and hemoglobin levels) were significantly different between cases and controls. Enemas were not used in the enrolled participants.

**Figure 2:**
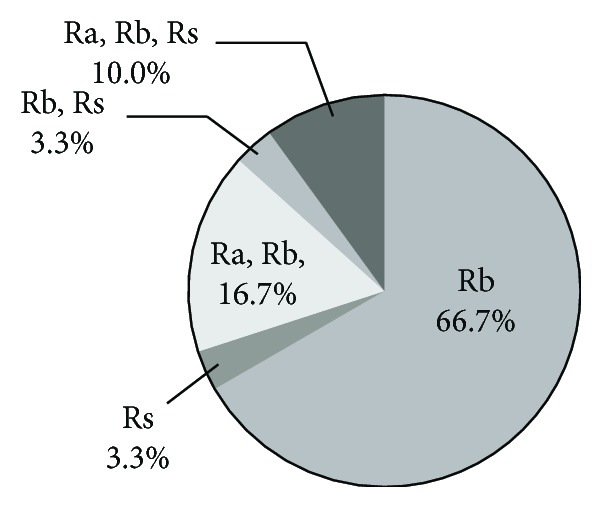
Pie chart depicting the location of rectal ulcer in 30 of 38 patients with acute hemorrhagic rectal ulcer syndrome (in 8 cases, rectal ulcer location was not described in endoscopy report). Rs: rectosigmoid; Ra: rectum above the peritoneal reflection; Rb: rectum below the peritoneal reflection.

More than half of the rectal ulcer was located in Rb (Figure 2). We performed biopsy in eleven patients. The histological findings of all these patients were nonspecific and did not show any finding suggesting SRUS or IBD.

### 3.2. Density Case-Control Analysis for the Identification of Risk Factors for AHRUS

In bivariate logistic regression analyses, age, hospitalization, antithrombotic drug use; the comorbidities of ischemic heart disease, cerebrovascular disease, and chronic kidney disease under hemodialysis; and the laboratory findings of white blood cell count, hemoglobin, and serum albumin values were significantly associated with AHRUS.

In multivariate logistic regression analyses, hospitalization (adjusted OR 15.65, 95% CI 2.25–108.9), antithrombotic drug use (adjusted OR 12.05, 95% CI 1.53–94.4), and one gram decrease of serum albumin (adjusted OR 0.11, 95% CI 0.02–0.52) were significantly associated with AHRUS. On the other hand, neither of age, the indicated comorbidities were not significant risk factors (Table 2).

### 3.3. Outcome and Long-Term Survival of Rectal Bleeding

Endoscopic treatment for attempted control of bleeding (clipping or band ligation) was performed in 8 of the 38 patients (21%), as listed in Table 3. Rebleeding occurred in two patients and was treated successfully with reclipping. Sixteen of the 38 patients (42%; 95% CI 28–58%) needed blood transfusion. All patients received hydration and restriction of oral intake. We did not use sucralfate enema in any patient.

**Figure 3:**
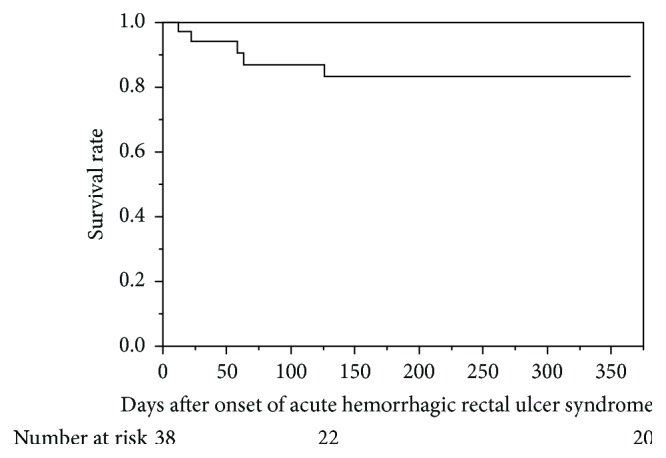
Kaplan-Meier estimates after onset of acute rectal hemorrhagic rectal ulcer. 17 percent of patients with acute hemorrhagic rectal ulcer syndrome (AHRUS) died of non-AHRUS problem in one year.

Survival analysis showed that 17% of patients died within one year after their rectal bleeding episode from causes not related to their AHRUS (Figure 3). These results collectively indicated that AHRUS could be an unfavorable prognostic condition, and the preventive intervention based on the risk factors would be necessary.

Among 33 survivors at one year, follow-up colonoscopy at least 30 days after rectal bleeding was performed in four patients; complete rectal ulcer healing was confirmed in all these patients.

## 4. Discussion

This study is the first case-control study that we are aware of about risk factors for AHRUS. We found that hospitalization, antithrombotic drug use, and hypoalbuminemia were significant risk factors for developing AHRUS. Seventeen percent of AHRUS patients died within one year of causes not related to the AHRUS.

Rectal ulcers with massive hemorrhage in critically ill patients have long been recognized [6, 14, 15], but the infrequency and fatal clinical course has precluded clinical studies about its risk factors and establishing this condition as an independent clinical disease. Recently, however, rectal ulcers with massive hemorrhage have been recognized as an emerging clinical entity, AHRUS [3, 6, 7, 16].

Previous observational studies have reported the characteristics of AHRUS patients, such as older age, immobility, antithrombotic drug use, and comorbidities such as diabetes mellitus, coronary artery diseases, cerebrovascular attacks, sepsis, liver failure [3], hypoalbuminemia [17–19], and chronic renal failure with hemodialysis [15]. The characteristics of cases in our research were compatible with those in observational studies and supported etiological assumptions except for older age [17–20]. Like others [6, 7], we found that AHRUS patients often require hemostatic procedures and blood transfusion. We did not use sucralfate enemas in our patients, although a case series [21] reported it effective.

Several studies have shown that serum hypoalbuminemia can be an independent risk factor for decreased microperfusion and pressure ulcers because albumin helps maintain oncotic pressure and vascular refilling [22–24]. Our results were compatible with the results of the previous observational study, which suggested that hypoalbuminemia and high blood urea nitrogen levels were risk factors for lower gastrointestinal bleeding, mainly due to ischemic colitis and rectal ulcer in critically ill patients [19].

It seems reasonable that immobility during hospitalization, hypoperfusion of local rectal blood flow in the elderly, and hypoalbuminemia could lead to the formation of rectal ulcer; previous epidemiological studies suggested that these could be risk factors of SRUS or stercoral ulcer [2, 9, 10]. Our endoscopic findings indicated that more than 90% of rectal ulcers were located in Rb, which implied specific local vascular flow disturbance.

Baseline blood flow in the rectal mucosa, measured by laser Doppler flowmetry, has been found significantly below normal in patients with SRUS [9] or AHRUS [25], and the blood flow is significantly reduced in the horizontal supine position at bed rest. Baroreceptor-mediated vasoconstriction in hypovolemic conditions [26] or in bedridden, elderly, or hospitalized patients has been reported to cause ischemic proctitis [27].

Although ischemia might be involved in both SRUS and AHRUS, the pathogenesis of the two clinical entities seems different. Comparing with patients with SRUS associated with fecal evacuation disorders [10], the higher prevalence of AHRUS among immobilized older patients with cardiovascular risks [6, 7] suggests that nutrient blood flow is disturbed in AHRUS, rather than there is local or direct pressure in the rectum as in SRUS.

Our study has limitations and strengths. First, we did not assess the possible role of constipation or overactivity of the anal sphincter in causing rectal ulceration or ischemia because of retrospective design. Second, there could be selection bias by excluding subjects with missing laboratory values; this bias might have an effect toward the null on the odds ratio of hospitalization for AHRS. Despite these factors, our study was valuable because this was the first density case-control study in Japan to explore the comprehensive risk factors for the occurrence of AHRUS and patients' survival after AHRUS.

## 5. Conclusions

Hospitalization, antithrombotic drug use, and hypoalbuminemia were significant risk factors for AHRUS. Knowledge of these risk factors could make clinicians more alert to the possibility of AHRUS being the cause of lower gastrointestinal bleeding, to take measures to prevent it and to perform proctoscopy or colonoscopy promptly to diagnose it. Onset of AHRUS has an unfavorable prognosis.

## Figures and Tables

**Table 1:** Patients' characteristics and bivariate analyses.

Variable	Cases	Controls	*P* value
Age (years), mean (SD)	76 (12)	60 (15)	<0.0001
Sex, male, *n* (%)	18 (47.4)	62 (50.4)	0.85
Hospitalization, *n* (%)	32 (84.2)	14 (11.4)	<0.0001
Hospitalization period (day), mean (SD)	19.0 (24.4)	1.0 (4.9)	<0.0001
Usage of antithrombotic drugs, *n* (%)	25 (65.8)	16 (13.0)	<0.0001
*Comorbidity*			
Hypertension, *n* (%)	23 (60.5)	45 (36.6)	0.014
Diabetes mellitus, *n* (%)	10 (26.3)	19 (15.5)	0.15
Ischemic heart disease, *n* (%)	13 (34.2)	5 (4.1)	<0.0001
Cerebral vascular disease, *n* (%)	17 (44.7)	8 (6.5)	<0.0001
Under hemodialysis, *n* (%)	4 (10.5)	3 (2.44)	0.054
Malignancies, *n* (%)	6 (15.8)	30 (24.4)	0.37
*Laboratory findings*			
Serum albumin (mg/dl), mean (SD)	2.67 (0.60)	4.09 (0.52)	<0.0001
Serum Cr (mg/dl), mean (SD)	1.35 (1.50)	1.16 (2.21)	0.63
Serum AST (U/l), mean (SD)	26.0 (14.6)	22.8 (9.9)	0.13
Serum ALT (U/l), mean (SD)	25.3 (19.9)	23.3 (21.8)	0.62
White blood cell count (cells/*μ*l), mean (SD)	8013 (4757)	5959 (2047)	0.0002
Hemoglobin (g/dl), mean (SD)	9.51 (2.09)	12.7 (2.06)	<0.0001
Platelet count (thousand cells/*μ*l), mean (SD)	247.3 (132.4)	236.7 (90.8)	0.58

SD: standard deviation; AST: aspartate aminotransferase; ALT: alanine aminotransferase.

**Table 2:** Bivariate and multivariate logistic regression analyses.

Variable	Crude OR	95% CI	*P* value	Adjusted OR	95% CI	*P* value
Age	1.10	1.06–1.14	<0.0001	1.03	0.96–1.11	0.36
Hospitalization	41.52	14.8–116.8	<0.0001	15.65	2.25–108.9	0.006
Antithrombotic drug use	12.86	5.49–30.13	<0.0001	12.05	1.53–94.4	0.018
*Comorbidity*						
Hypertension	2.66	1.26–5.61	0.0093	0.51	0.08–3.18	0.47
Diabetes mellitus	0.13	0.82–4.68	0.141	1.33	0.12–14.8	0.82
Ischemic heart disease	12.27	4.01–37.53	<0.0001	8.44	0.89–80.3	0.063
Cerebral vascular disease	11.64	4.45–30.40	<0.0001	2.41	0.42–13.7	0.32
On hemodialysis	4.71	1.00–22.05	0.049	0.66	0.01–33.5	0.84
*Laboratory findings*						
White blood cell count	1.26	1.09–1.46	0.0004	1.24	0.94–1.63	0.11
Hemoglobin	0.53	0.43–0.66	<0.0001	0.97	0.59–1.60	0.9
Serum AST	1.02	0.99–1.05	0.145	0.98	0.92–1.05	0.64
Serum albumin	0.036	0.01–0.10	<0.0001	0.11	0.02–0.52	0.006

OR: odds ratio; AST: aspartate aminotransferase.

**Table 3:** Endoscopic hemostatic procedure.

Procedures and rebleeding rate	Cases
Hemostatic procedure, *n* (%)	8 (21)
Band ligation, *n*	2
Clipping, *n*	6
Rebleeding after hemostatic procedure, *n* (%)	2 (25)

## Data Availability

The data used to support the findings of this study are available from the corresponding author upon request.
